# Genome-wide association analysis of Framingham Heart Study data for the Genetics Analysis Workshop 16: effects due to medication use

**DOI:** 10.1186/1753-6561-3-s7-s52

**Published:** 2009-12-15

**Authors:** Treva K Rice, Yun Ju Sung, Gang Shi, C Charles Gu, DC Rao

**Affiliations:** 1 Division of Biostatistics, Washington University School of Medicine, Box 8067, 660 South Euclid Avenue, St Louis, Missouri 63110 USA; 2 Department of Psychiatry, Washington University School of Medicine, Box 8067, 660 South Euclid Avenue, St Louis, Missouri 63110 USA; 3 Department of Genetics, Washington University School of Medicine, Box 8067, 660 South Euclid Avenue, St Louis, Missouri 63110 USA

## Abstract

Problems associated with medication use and the consequent effects on genome-wide association analyses were explored using the Genetic Analysis Workshop 16 Problem 3 data. Lipid phenotypes were simulated in the Framingham Heart Study using several measured variables including causal genes (based on a 500 k SNP panel), smoking, dietary intake, and medication usage. We report a sensitivity analysis of how medication use (which artificially alters lipid values) affects association inferences. Associations were performed for LDL-c under seven different correction schemes: 1) ignore medication use entirely (no correction) and adjust for covariates; 2) delete medicated subjects then adjust for covariates; 3) include medication use (Yes/No) as a covariate during covariate adjustments; 4) correct raw values using clinical trials information then adjust for covariates; 5) correct raw values using the actual simulation protocol ("truth") then adjust for covariates; and 6-7) over-corrections (add arbitrary values) then adjust for covariates. Results indicate that failure to properly correct for medication usage can profoundly affect the heritability, and hence the association results. The empirical results yielded one genome-wide significant locus on chromosome 22 (RS2294207), consistent with the simulation protocol. This signal was detected under all schemes that corrected the raw values (clinical trials, simulation protocol, or over corrections), but was not detected under the first three adjustment schemes (ignore medication use, delete medicated individuals, use medication status as covariate). In summary, we confirm that failure to properly account for medication usage can have a profound impact on genetic associations.

## Background

In addition to the multiple technical challenges in analyzing genome-wide association (GWA) data, there remain some basic questions about how to properly derive the phenotype when measured traits are artificial for some (but not all) individuals, e.g., due to medication use. How should adjustments for such confounders be performed? For age, which is known to inflate the resemblance among family members, the general procedure is to remove the effect using a regression procedure and retain the residual. However, medication is a different kind of confounder that artificially alters trait values, but only in treated individuals. One could consider drug use (e.g., yes/no) similar to age by using it as a covariate in the regression procedure. This minimizes mean differences in lipid levels between users and non-users but does not recover the original (unmedicated) values. In the context of linkage studies, Cui et al. [1] showed that either ignoring the fact that some individuals were medicated or excluding them led to a reduced ability to detect genetic effects. Epstein et al. [2] considered this to be a data censoring problem. Their variance-components linkage model was based on the regression method of Tobin [3] for analysis of censored normal data under the assumption of independent observations. Basically, it allowed for latent values to be more extreme than observed. Analysis of simulated data that had been corrected using the censoring model led to unbiased parameter estimates and improved type I error rates. However, failure to correct for censoring led to underestimates of the mean effects (in this case the genetic effects). This suggests that simply adding an appropriate constant to treated (censored) data is appropriate (see linkage studies [1,4-6]). To address these concerns in the context of association studies, we analyzed simulated lipid data (the "truth" is known) in which some subjects were medicated and others were not. In particular, this investigation looks at the sensitivity of alternative methods of medication correction and their impacts on association results.

## Methods

### Study design and simulation of data

We used the Genetic Analysis Workshop (GAW) 16 Problem 3 simulated data, which is based on the Framingham Heart Study. Lipid data were simulated under a model that used the actual pedigree structures and sample sizes of the Framingham data (6,476 subjects in 942 pedigrees distributed among three generations) across three time points that were 10 years apart. During pedigree construction the family members' relative ages were calculated at a given exam, and then ages were assigned for everyone at three fixed, 10-year intervals. There was no drop-out and no missing data.

Genotypes were derived from the Affymatrix GeneChip^® ^Human Mapping 500 k Array Set (approximately 550,000 SNPs). While the real marker data was used, lipid phenotypes were simulated under a model with known major genes and polygenes, as documented in Kraja et al. in this issue [7]. In this report, we concentrate on low-density lipoprotein-cholesterol (LDL-c), which is the trait that had the largest medication effect. LDL-c was influenced by six major genes with locus-specific heritabilities ranging from 1% to 0.1%. The polygenic heritability was 52% (1,000 genes), and the polygenic and locus-specific effects together accounted for 55% of the variation. Medication (yes/no) effects were modeled as a pharmacodynamic process. When simulated lipid values were checked, individuals in the upper tail of the distribution (2% at Visit 1 and 15% at Visit 3) were classified "medicated" and responders were assigned a 30% decrease in values. Smoking and diet also had effects on the simulated traits.

### Data adjustments

Our analysis plan consisted of seven different correction schemes (see Table 1). Scheme 1: we ignored the fact that some of the individuals were medicated. Scheme 2: we recognized that lipid values were artificial for medicated subjects by assigning their trait values to be missing. Scheme 3: medication use was treated as a binary covariate (yes/no) during covariate adjustments (see below). For the remaining schemes (4-7) we applied a correction to the raw data to try to recover the values that would have been seen if there had not been any lipid-lowering drugs. Scheme 4 (clinical trials): involved a correction for the effects of HMG-CoA reductase inhibitors (statins) as outlined in the study by Wu et al. [6]. Wu et al. [6] derived correction factors by using the data from 32 clinical trials (with over 19,000 participants) that looked at long-term effects (greater than 8 weeks) of anti-hyperlipidemic medications. Weighted-average absolute changes in lipid values were calculated by medication and ethnic groups. In the current investigation, the correction simply involved adding the tabled value to the reported LDL-c trait values (*X *+ 48.1 mg/dl). Scheme 5 ("truth"): was based on the protocol used in the data simulations [7]. Because an individual's original value was reduced by 30% to create the medicated responder value, we reversed the adjustment as shown in Table 1 [*Y *= *X*/(1-0.3)]. Schemes 6 and 7: we applied two over-adjustments by adding arbitrary constants (*X *+ 75 mg/dl for Scheme 6 and *X *+ 100 mg/dl for Scheme 7).

**Table 1:** Analysis variables, corrections and total heritabilities

LDL-c			Heritabilities	
	
Scheme	Method of adjustment	Correction	Visit 1	Visit 3
1	No correction, ignore medication status	None	43.8%	25.2%
2	No correction, assign medicated values as missing	None	44.3%	33.5%
3	No correction, treat medication (Y/N) as covariate	(Covariate)	43.6%	30.1%
4	Use clinical trials data to correct LDL^a^	*X *+ 48.1 mg/dl	46.6%	47.9%
5	Use GAW16 simulation protocol to correct LDL^b^	*X*/(1 - 0.3)	46.6%	50.1%
6	Over-correct LDL (1)	*X *+ 75 mg/dl	46.6%	50.0%
7	Over-correct LDL (2)	*X *+ 100 mg/dl	45.7%	48.5%

^a^See Wu et al. [6] for details.

^b^Original values were reduced by 30% [*X *= *Y *- 0.3(*Y*)].

Each of the (raw or corrected) LDL-c traits was then adjusted for several covariates using a stepwise regression method. The covariates included a polynomial in age (age, age^2^, age^3^, age^4^, age^5^, and age^6^), smoking (yes/no), and diet (intake per day). Additionally, medication use (yes/no) was a covariate for Scheme 3. In summary, a given measure was regressed on these covariates, separately in males and females, using in a stepwise procedure. Only significant terms (5%) were retained to compute the residual from this regression. The residual was standardized to a mean of zero and a variance of one. The entire correction and adjustment procedure was carried out separately for each of Visits 1 and 3 data, leading to seven Visit 1 and seven Visit 3 variables, as outlined in Table 1.

### Association analysis

Variance-components models as parameterized in the computer programs QTDT [8] and Merlin [9] were used to perform heritability and association analyses. Both were maximum likelihood procedures estimating parameters under two contrasting models, with the difference in (minus twice) the likelihoods being distributed as a χ^2 ^based on 1 degree of freedom. For heritability, a model with only residual environmental (*e*) effects is compared to another having both the environmental and a polygenic heritability (*e *+ *g*) component. The likelihood comparison tests the null hypothesis that the heritability is zero. The association analysis is equivalent to a non-TDT family-based method that contrasts the environmental plus polygenic model (*e *+ *g*) with one in which the environmental, polygenic and association component is included (*e *+ *g *+ *a*). The comparison tests the hypothesis that the association component is zero.

## Results

For covariate adjustments, age was the primary predictor, accounting for up to 10% of the variability. Additional covariates of smoking and diet did not substantially alter the estimates.

**Figure 1:**
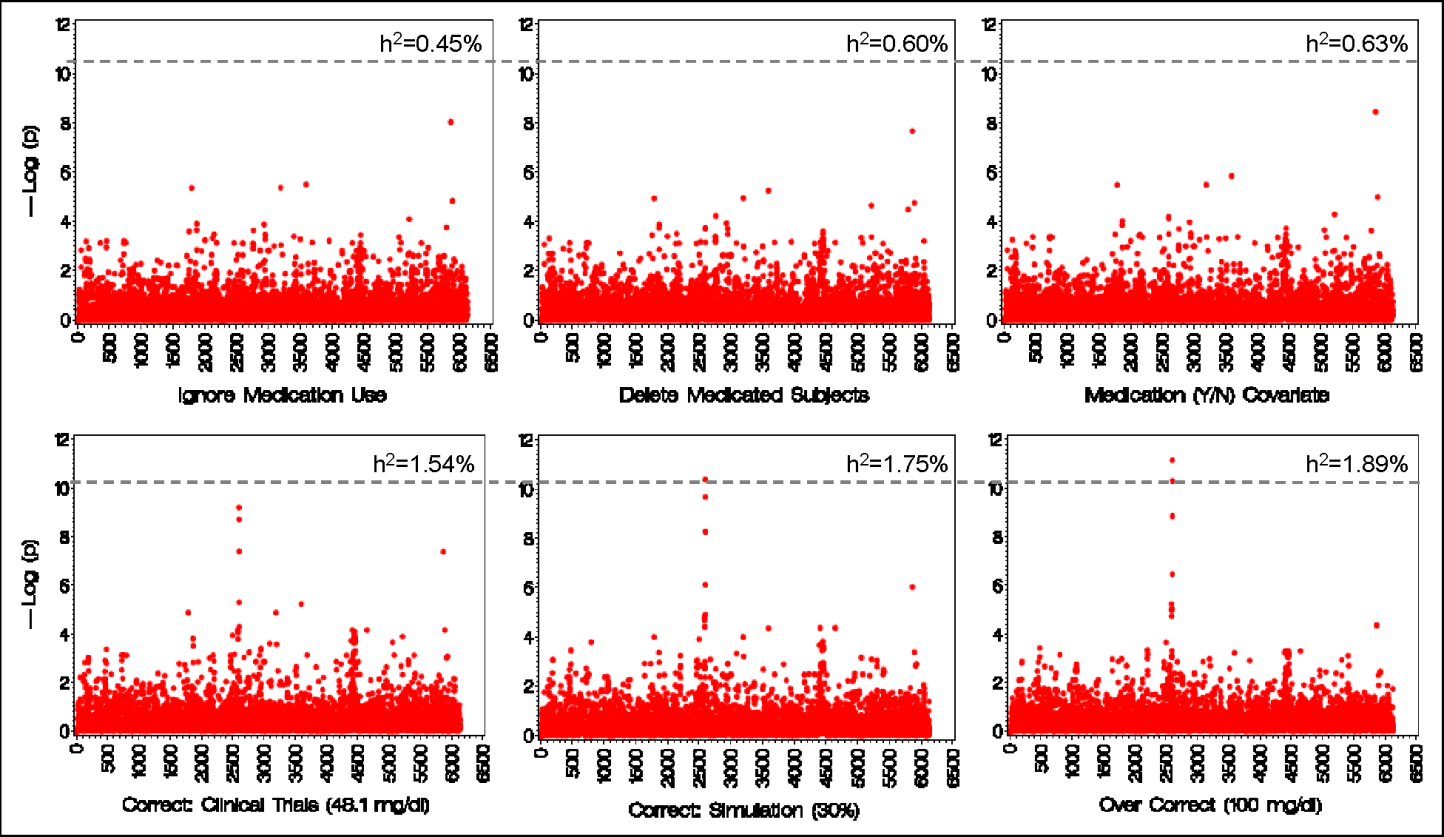
**Association results for Visit 3 LDL on chromosome 22**. Within each panel the vertical axis is -log_10_(*p*) for the association test and the horizontal axis represents the SNP markers (numbered sequentially). Panels on the top row (left to right) are ignore medication use, delete medicated subjects, and medication (Y/N) covariate. Panels on the bottom row are (left to right) corrections based on clinical trials data, the simulation procedure, and an over-correction. The dotted reference line represents the "true" -log(*p*) at the disease locus (RS2294207), with locus-specific heritabilities at the disease locus shown in each panel.

Heritabilities are in Table 1. There is a trend for the heritabilities to be larger with more appropriate medication correction. At Visit 3, the heritability goes from 25% for no correction to ~50% when the "true" correction is applied. There is little difference across adjustments for Visit 1. The Visit 3 association results for the first three adjustments (top row of Figure 1) show no genome-wide significant associations, but for the remaining adjustments (bottom row), there was a cluster of results at marker SNP_A-1908298 (RS2294207). The locus-specific heritability at RS2294207 is shown in each panel of Figure 1 (ranging from about 0.5% to over 1.5%). Based on the answers [7], this is the marker simulated to have the largest effect.

## Discussion

The purpose of this study was to perform a sensitivity analysis to determine how medication use, or rather how we adjust for medication use, affects genetic inferences in association analyses when the "true" answer is known. As expected, our results confirm that appropriate adjustments provide more accurate results. However, an important message is that some commonly used methods to correct for treatment may yield misleading results.

The "true" results were derived by pre-correcting the raw LDL-c values using the simulation protocol and then performing typical covariate adjustments. A series of analyses were performed under a variety of correction scenarios and compared to the truth. As expected, ignoring medication (Scheme 1) use biased the results for Visit 3 data (heritability was half of the true value of about 50%). However, two commonly used adjustment methods also were biased (Scheme 2, dropping medicated subjects or Scheme 3, treating medication use as a binomial covariate). While both of these methods slightly improved the signals as compared with doing nothing (heritabilities increased from 25% to ~30%) they did not yield "true" results. And, in fact, the association signal was completely missed under all three of these schemes. Removing medicated individuals decreases both the sample size and the power, but even more importantly these are the very individuals who provide the best evidence for genetic effects - the original upper tail! The alternative method that used medication as a binomial covariate (Scheme 3) assumes that the original unmedicated values of both the treated and untreated groups are similar. However, this is not the case (in the simulation or in real life) because only those individuals with sufficiently high original values are treated. In other words, this method tries to equalize the means across treated and untreated groups, when in fact the means by definition cannot be the same.

For the remaining schemes we found that any of the methods that attempted to recover the original (untreated) levels prior to covariate adjustment worked fairly well in terms of finding the "true" answer. In this dataset, the clinical trials method (Scheme 4) yielded a slight under-adjustment, while adding arbitrary values of 75 mg/dl (Scheme 6) or 100 mg/dl (Scheme 7) led to slight over-adjustments, as shown in the magnitudes of the heritabilities and the association signals. This strongly suggests that independent data such as that from the clinical trials literature can be used to provide correction factors that, while they are not exact, seem to be close enough to recover signals that otherwise would have been missed. However, in some situations more accurate approaches may be required. For example, information on not only the mean (as used here) but also on the variation is available in the clinical trials literature which could be used to account for individual variation in treatment effectiveness. In addition to genes, "environmental" factors such as compliance levels or longitudinal information about treatment also may influence individual variation. Although beyond the scope of the current study, all of this information could be incorporated using a mixed effects model, through multiple imputation, or through a regression that takes into account censored information such as the Tobit model (see [2,3]).

In the last two to three years, GWA studies have been making great strides in identifying common polymorphisms associated with disease risk. For example, over 50 novel loci have now been detected that modify the risk for type 2 diabetes and cardiovascular disease [10] and over 20 new loci have been implicated in breast cancer [11]. However, each of these loci accounts for only a very small percent of the variance, typically much less than 1%, with the sum of all of loci still not accounting for all of the known genetic variance [12,13]. Consequently, a major problem that we face today is to identify and locate this "missing heritability". Currently, researchers are marshalling their efforts toward this end using a variety of tools, technologies, and methods. For example, large samples are being created through cooperative agreements among smaller studies (e.g., the CARe and CHARGE consortia) in order to increase power. Others are integrating gene expression data, or extending GWA studies, to look at non-traditional markers such as copy-number variations. Certainly, these are good places to start looking. However, attention to the phenotype itself should not be neglected. As the current study shows, treatment acts as noise that overpowers the small signals produced by individual loci and prevents their detection. We have shown that how one corrects for this confounding factor can be crucial.

## Conclusion

In summary, these results show that medication use can have a significant effect on genetic analysis. Careful consideration in how one corrects for treatment is important in the case of association studies in which very small effects are expected as compared to the relatively large degree of noise that is produced when values are modified due to treatment effects.

## List of Abbreviations

GAW: Genetic Analysis Workshop; GWA: Genome-wide association; LDL (LDL-c): Low-density lipoprotein-cholesterol; QTDT: Quantitative transmission-disequilibrium test; SNP: Single-nucleotide polymorphism; TDT: Transmission disequilibrium test

## Competing interests

The authors declare that they have no competing interests.

## Authors' contributions

CCG carried out quality control analyses of the genotypes. GS extracted the data and set up genotype and map files in the appropriate formats. YJS performed association analyses. DCR participated in the design and coordination of the study. TKR conceived the study, derived all medication correction phenotypes, performed final formatting of analysis datasets, collated all results, and drafted the manuscript. All coauthors approved the final manuscript.
